# Developing a shared pedagogical statement for a university psychology teaching department: is it possible?

**DOI:** 10.1080/00049530.2026.2635899

**Published:** 2026-04-01

**Authors:** Lisa Lole, Michelle Gossner, Paul Duckett, Cassandra K. Dittman, Sharleen Keleher

**Affiliations:** a College of Psychology, School of Health Medical and Applied Sciences, Central Queensland University, Bundaberg, Australia; b School of Psychology, The University of Queensland, Brisbane, Australia

**Keywords:** Higher education, pedagogy, psychology, teaching statement, teacher values

## Abstract

**Objective:**

The Australian Psychology Accreditation Council (APAC) requires higher education providers to have a pedagogical statement that describes their collective approach to curriculum design and delivery. The current study describes the process taken by one regional Australian university when attempting to develop such a statement.

**Method:**

A multi-stage, embedded mixed-methods approach, incorporating student feedback, staff reflexivity, and dialogic methods were used. Participants’ accounts of their personal pedagogy and practices were expected to inform the development of a coherent pedagogical statement, through an inductive approach to thematic analysis.

**Results:**

Despite participants’ professional experience and content expertise, we were unable to develop a traditional pedagogical statement due to their limited awareness of the theoretical framework/s that inform their practice. Instead, we developed a values-based teaching statement that described what the team considered professionally important. Participation in the project was reported to improve confidence in discussing and conceptualising pedagogy among staff.

**Conclusion:**

Values underpin teacher philosophies and practices and may encourage further reflection, exploration, and communication of relevant pedagogical concepts, including teacher philosophies and practices. Findings from the current research have important implications for the development of pedagogical culture locally, as well as for accrediting bodies, universities, and individual educators more broadly.

## KEY POINTS

**What is already known on this topic:**

1. An effective pedagogical approach is widely recognised as a critical contributor to positive education outcomes.
2. Collective pedagogies for teaching teams have also been argued to have positive benefits for students and educators.
3. Psychology is a diverse discipline, in which students and teaching professionals may adhere to equally-diverse philosophies and practices.

**What this topic adds:**

1. Understanding about effective methods and prompts that can be used to generate conversations about teaching philosophies and practices, as well as encourage staff confidence and competency in discussing pedagogy.
2. Consistent with previous research, most higher education staff do not hold formal education qualifications and have difficulty discussing their personal pedagogy. Encouraging them to reflect upon and discuss their values with colleagues appears to be a positive step towards their professional development in this regard.
3. This topic may help to identify the commonalities and diversities that unite and strengthen teaching teams, leading to reduced confusion and division among staff.

Pedagogy is broadly defined as the theories and methods concerned with learning and teaching practices. An effective pedagogical approach is widely recognised as critical in enabling teacher and development supporting student learning (Bridoux et al., 2023; Vreekamp et al., 2023; Waring & Evans, 2015). Previous literature has highlighted the benefits of individual teachers having a personal pedagogical philosophy reflecting their learning and teaching philosophy and practice, which are typically guided by individual educators’ values, beliefs, knowledge, and experiences, as well as individual, cultural, and socioeconomic factors (Patfield et al., 2022).

Shared, group-level pedagogies have also been argued to effectively guide professional development and complement individual pedagogical approaches (Conway & Andrews, 2016). Like individual pedagogies, collective pedagogies summarise the “what”, “how”, and “why” of teaching, but from the perspective of a teaching team, profession, or discipline (Gurung et al., 2008; Shulman, 2005). The aim of such is to describe the collective approach to teaching and learning, and provide a cohesive representation of teaching practices and philosophies within a shared framework. Such statements can promote a sense of place, organisation, direction, empowerment, satisfaction, and morale among staff (Chick et al., 2012). Group-level pedagogical approaches at a program or degree level may also support student engagement and reduce attrition, through the provision of coherent, clear, and streamlined learning and teaching experiences (Kahu, 2013; Picton et al., 2018; Ulferts, 2019).

There is considerable literature on how to implement group-level pedagogies that target key learning and teaching strategies in a top-down manner (e.g., Cranney et al., 2022; Douglass et al., 2023; Fields et al., 2021). However, such a prescriptive approach to group-level pedagogical statements may prove challenging in circumstances where staff do not have formal training in education or in teams where pedagogical culture is underdeveloped (Admiraal, 2024; Halse, 2011; Weimer, 2008). Variation in pedagogical approaches exists both between and within academic disciplines (Huber & Hutchings, 2005; Neumann, 2001) and challenges may also arise if there is diversity in learning and teaching practices across the curriculum or in worldviews among staff (Ion & Iucu, 2014; Wieser, 2016; Nind & Lewthwaite, 2018). The task of synthesising the core values, goals, and worldviews across such diversity warrants attention (Van Schaik et al., 2019); however, research in this space is limited.

## Context

Psychology is a broad discipline that focuses on scientifically understanding the human mind and behaviour, through various ways of knowing that span a wide spectrum of ontologies, epistemologies, and methodologies. Students of psychology pursue diverse career and study pathways (Collisson & Eck, 2022; Roscoe & McMahan, 2014). This diversity can lead to confusion and division among staff about the core purposes and principles that unify the field (Peden & VanVoorhis, 2009). While many disciplines have unifying signature pedagogies, in psychology, these are limited to the sub-discipline level (Baltrinic & Wachter Morris, 2020; Day & Tytler, 2012; Peden & VanVoorhis, 2009). In Australia, the Australian Psychology Accreditation Council (APAC) regulates the content and outcomes of psychology higher education. APAC requires psychology education providers to specify, “a coherent educational pedagogy that informs the documented program design and delivery” (Australian Psychology Accreditation Council [APAC], 2019, p. 9).

At our university, the department of psychology has 28 permanent staff members. Most staff are employed on continuing rather than fixed-term contracts. The university offers five accredited undergraduate psychology courses, two accredited postgraduate training programs, and one non-accredited postgraduate course. Our university has a main campus in regional Queensland and satellite campuses in metropolitan and regional locations nationwide, meaning that staff and students do not always share the same physical space. There is widespread use of both online and blended learning delivery modes, with synchronous and asynchronous engagement delivery options available to students. Many students are from low socioeconomic backgrounds, are first-generation university students (i.e., the first in their family to pursue higher education), mature age, and/or live in regional, rural, and remote areas.

## The current research

Dialogic and negotiated approaches to knowledge have been shown to foster collegial relationships and promote new understandings of pedagogy (Nind, Kilburn, & Luff, 2015; Nind, Kilburn, & Wiles, 2015, 2016, 2018). Student feedback, including feedback that is provided anonymously through satisfaction surveys, is a source of valuable insight into learning and teaching practices at our regional university, where many students study online (meaning that opportunities to hear from students is arguably more limited compared to on-campus settings; Sharma & Shree, 2023). Moreover, research suggests that professional development activities that require active staff participation and collaboration to discuss, develop, evaluate, and improve practices, are highly valued by staff participants (Hargreaves & O’Connor, 2017; Vreekamp et al., 2023). Our study developed and piloted a framework to create a cohesive group-level pedagogical statement for our university psychology department. The framework used student feedback, staff reflexivity, and dialogic methods (including professional conversations) as tools to enable reflection and expression among staff regarding their approaches to teaching and learning practices. The study sought to address the following research questions:

1. Can a framework that incorporates student feedback, staff reflexivity, and professional conversations be used to formulate a discipline-relevant, group-level pedagogical statement?
2. What barriers and enablers are faced when applying the framework?
3. What is the impact of engaging with implementation of the framework on staff confidence and their perceived capacity to reflect on their approach to teaching and learning?

## Materials and method

### Research design

**Figure 1.**
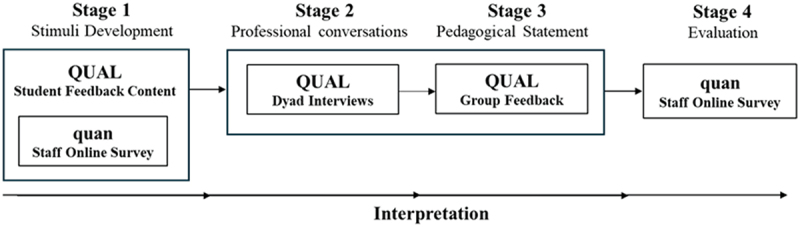
Methodologies used in the multiple phases of the research.

Our project employed mixed methods in a multistage, embedded research design, with more weight placed on the qualitative data (Creswell et al., 2003). There were four sequential stages, apart from Stage 1, where data were collected and collated concurrently (see Figure 1). Being teachers in the department, the research team had “insider” status in the inquiry (Costley et al., 2010; Kemmis, 2009; Loughran, 2013; Siemens, 2005). The study’s protocol was approved by CQUniversity’s Human Research Ethics Committee (clearance number: 23,367).

QUAL = qualitative (more weight given); quan = quantitative (less weight given).

### Participants, materials, procedure, and data analysis

#### Stage 1 – developing stimuli for professional conversations

The aim of this stage was to better understand the baseline for our colleagues’ teaching and learning practices and views on teaching. It comprised the following two forms of data.

##### Student feedback

During the last three weeks of each 12-week term, students at our university are invited to evaluate their units through a student experience survey. Invitations to complete these evaluations are sent from the university to each students’ email account. This survey asks students to provide anonymous numerical satisfaction ratings for specific domains and the unit overall, as well as written open-response feedback regarding the best aspects of the unit and areas for improvement. While the quantitative data provides data on whether students are satisfied with their learning experience, the open-response questions give student the opportunity to provide feedback on aspects of their learning experience and interactions with their teachers that are relevant to them, rather than on the pre-determined domains in the satisfaction survey. Thus, the qualitative data was used instead of the quantitative data as it provided richer insights into learning and teaching issues relevant to students. We used stratified sampling to select 11 psychology units from Term 2, 2021, ensuring units in all year levels of psychology coursework degrees were represented. This resulted in a sample of 3828 individual comments from six bachelor’s, two honours, and three master’s degree units. The open response data for the selected units were openly coded, and interpretive content analysis was used to inductively form themes (Drisko & Maschi, 2016). Themes were turned into four short statements and used as discussion prompts for professional conversations in Stage 2.

##### Online staff survey

An email invitation to complete a 22-item baseline survey was sent to all psychology teaching staff employed with the university on an ongoing basis (*N* = 28). Fifteen staff completed the survey. Staff were asked to answer structured and open-text questions on their employment and education history, teaching practices, and their approach to teaching and learning. Quantitative data was used to understand some of the baseline characteristics of teaching staff, through examination of descriptive statistics, which helped inform the development of questions and discussion prompts for Stage 2. Thematic analysis of open-text data helped inform the statement development in Stage 3 (Braun & Clarke, 2022).

#### Stage 2 – professional conversations

**Figure 2.**
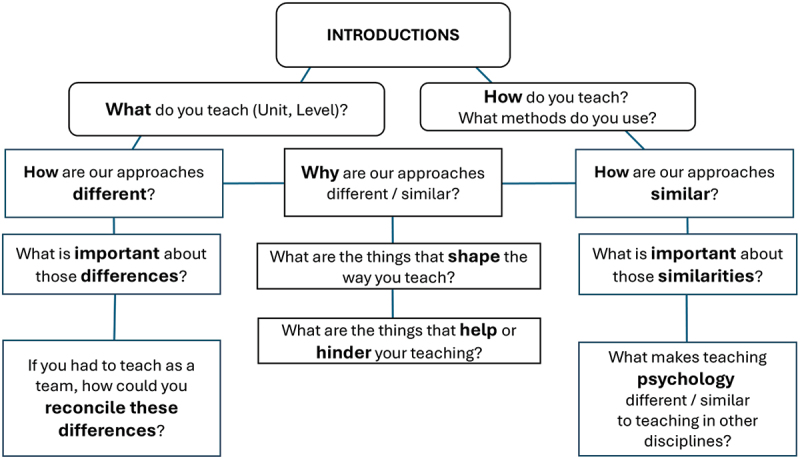
Stage 2 dialogic prompts for professional conversations.

Eight staff who expressed their interest in participating via the Stage 1 survey were grouped into four pairs for professional conversations. Pairs were purposely formed to maximise differences between participants (e.g., postgraduate – undergraduate courses; quantitative – qualitative research methods). There was a good representation of undergraduate and postgraduate staff, academic roles (research-focused, research-teaching balanced, and teaching-focused), and teaching areas within the discipline. Dyads participated in a professional conversation about teaching and learning, for approximately one hour via a videoconferencing platform (Zoom Communications, Inc.). Conversations were facilitated by a member of the research team (SK), who introduced the participants, reminded them about the aims of the project, and helped guide the conversation using questions and discussion prompts. Discussion prompts centred on the themes identified from student feedback in Stage 1 (see Figure 2). Apart from posing prompts and questions, SK did not take part in the conversation. Each conversation was recorded and transcribed verbatim by MG.

#### Stage 3 – pedagogical statement development

Reflexive thematic analysis of open-text survey response (Stage 1) and professional conversation data (Stage 2) were read, re-read, coded and consolidated into a set of core themes (Braun & Clarke, 2022). These themes reflected participants’ pedagogy, experiences, and approaches to teaching and learning. A draft values statement summarising these themes was presented to teaching staff at two separate department-wide staff forums, held online two months apart. Staff were invited to provide live feedback on the statement verbally in the forums, or in written form through a shared online document. Staff were encouraged to provide feedback after the forum via email to SK, or through the staff online survey in Stage 4. Staff were also invited to identify examples of how they enact or experience separate elements of the statement, via the shared online document.

#### Stage 4 – evaluation of roadmap impact

All psychology teaching staff (*N* = 28) were invited via email to complete a second online survey. Thirteen staff chose to participate. Staff were asked to respond to the same 22 survey items used in Stage 1, an open text feedback item asking for general feedback on the project, and an additional seven questions specifically designed to assess the direct impact of participating in the project. These seven items were rated on a 7-point Likert scale, ranging from −3 (negative impact) to + 3 (positive impact), with 0 indicating “no impact”. Mann-Whitney U analyses were used to compare Stage 1 and Stage 4 data, and Spearman rank correlation analyses to explore the impact of participation in project activities on participants’ professional development. While several of these analyses revealed several small to large effect sizes (see Table 1 and 2 of the **Online Supplementary Material**), they were also under-powered, limiting their generalisability. Thematic analysis was used for the open text feedback data (*n* = 10).

**Table 1.** Results and examples from the content analysis of student feedback in Stage 1 and the corresponding conversation prompts developed for Stage 2.

Content area	Student comment example	Conversation prompt
**Edu-tainment** A focus on enjoyment	“My lecturer makes the class fun”.	Do we need to entertain students?
**Keeping it real** The importance of practical skills and real-world examples	“It was good learning skills that I can use at work”.	Should we teach more practical and less theory?
**It’s personal** Focus on the attributes of the teacher	“My lecturer is really caring”.	How important is it for students to like their teacher?

**Table 2.** Themes extracted from the professional conversation data in Stage 2.

Theme	Description
Engagement	Staff valued *engaging* with students and exploring ways to strengthen students’ engagement with their learning.
Connectedness	Staff viewed a sense of *connectedness* with each other as important for both staff and students. They described the importance of connecting learning content and assessment with the real world.
Responsivity and reflexivity	Staff emphasised the importance of being *responsive* and cater to students’ needs in a *reflexive* and considered way.
Balance	Staff viewed *balance* as important to their teaching and learning. This included balancing administrative requirements, research and teaching in ways that supported their engagement with students
Passion	*Staff expressed passion* and love for the topics they taught and spoke about how they loved sharing their professional knowledge and interests with students.

## Results

### Stage 1

Themes formed through analysis of student feedback, examples of student comments from which they were derived, and corresponding Stage 2 prompts can be found in Table 1. Survey data revealed that only one participant reported having a formal postgraduate teaching qualification (a graduate certificate in tertiary education). Three participants indicated that they either did not have, or were “unsure” of whether they had, a clear approach that guided their teaching.

### Stage 2

A summary of themes formed from the professional conversations is provided in Table 2. These conversations played a critical role in shaping the final statement in Stage 3.

### Stage 3

The values-based teaching statement appeared to have emergent pedagogical elements. Specifically, participants expressed an empathetic, inclusive, and procedural approach to teaching and learning that was responsive to students’ needs and sought to cultivate feelings of belonging among students (Cigman, 2007; Li & Xue, 2023; MacLeod et al., 2019; Namanyane & Shaoan, 2021; Stentiford & Koutsouris, 2021; Wright, 2014). The value of “connecting students with their peers” reflected elements of collaborative pedagogy (Riebe et al., 2016).

However, while participants provided rich descriptions of their teaching and learning practice, values and goals, there was very limited reflection on teaching and learning theory and philosophy (i.e., few participants discussed their beliefs about how students learn and what learning means). Thus, we concluded that the development of a collective traditional pedagogical statement for our colleagues was not possible. Instead, we formed a teaching statement that reflected the shared values and practices participants considered meaningful, important, and relevant (Nind et al., 2016). The statement, along with examples of how each of the core values was enacted, can be found in Table 3.

**Table 3.** Our values-based teaching statement with descriptions and enacted examples.

Value	What this means	What this looks like
**Connecting with the world**	We connect learning and teaching to the world around us. We foster a mindset that uses psychology as a foundation for engagement with the world.	Using **analogies** or **stories** to help students understand difficult concepts. Using **case studies** and **examples** as the basis of assessment tasks or in-class activities so that content is applied / considered in the context of a real person and giving students an opportunity to **apply** skills and knowledge.
**Connecting with each other**	We build strong connections between staff and students to create safe, supportive learning environments, where expertise is shared and meaningful social bonds are made, where we can take risks, make mistakes, and grow professionally and personally, where people feel they belong and are valued.	Purposefully creating opportunities for students to engage with informal and formal **collaborative learning** activities. Connecting our students with **each other**; e.g., through support of the Student Psychology Society, campus catch-ups. Encouraging and supporting teaching staff to **try new things, share** their learnings with colleagues and to lead and participate in **special interest groups** that build our skills and knowledge.
**Teaching with purpose**	We teach a curriculum that is relevant, informed and evolves in a purposeful way. We use teaching practices that meet the diverse learning needs of our students and acknowledge our strengths. We use data, feedback and reflective practice to make informed decisions about strategies and approaches to teaching.	Engaging with **student strengths** to support each student’s learning; e.g., via peer-based learning. Designing our curricula to ensure we are **scaffolding** the knowledge, skills and competencies detailed in APAC accreditation standards, within and across our psychology courses. Engaging in **professional development** about effective teaching and learning practices with our diverse student cohort.
**Teaching for meaning and impact**	We teach to create opportunities, possibilities and pathways for our students. We teach to meaningfully impact the lives of our students so that they can positively impact their communities. We develop students’ knowledge and skills in psychology to prepare them for the range of professional environments they may enter.	Developing opportunities for students to work with **community stakeholders**. Supporting students to broaden their contacts with **potential employers** and **future career pathways**. Actively incorporating **First People’s perspectives** into our teaching. Building awareness of the relevance and currency of student skills for **real-world applications** and future **professional careers**.
**Driven by a passion for learning and teaching**	We draw on our passion for learning and teaching and for the discipline of psychology to build knowledge and skills in our students.	Staff talked about how they were inspired by some of the **transformations** that learning has on people’s lives; how they were invested in creating **memorable moments** and responding to students’ **diverse experiences, needs and ambitions**; and finally, how they strove to **harness the passion** of our students and staff to get through the difficult times and **celebrate** the successes.

### Stage 4

In open-text responses, most respondents reported that the project was “worthwhile” or “important”. Participation in the project reportedly gave staff an opportunity to reflect on their teaching practices and philosophy, to learn something new, and to have meaningful connections with their colleagues. One participant expressed regret at not being able to take part in the project to a greater extent, citing competing workload pressures as the cause of this. Another suggested that the time between the stages of the project should be shortened to enable better engagement with the process.

## Discussion

### Research question 1

While the incorporation of student feedback, staff reflexivity, and professional conversations did not facilitate the development of a traditional pedagogical statement, it did enable the formulation of a discipline-relevant, group-level, values-based teaching statement. The inability of staff to articulate pedagogical position, despite extensive teaching experience and content knowledge, is consistent with findings from previous research (Moreira et al., 2023; Van Schaik et al., 2018). Student feedback served as an effective stimulus to enable staff reflexivity in the context of dialogic professional conversations. While Nind and Lewthwaite (2018) chose to steer clear of pedagogical action research with staff (instead involving expert pedagogical leaders in their research), we actively engaged our colleagues who have disciplinary expertise in psychology. We interpreted the fact that they did not necessarily have pedagogical expertise in teaching and learning as the main reason for us not being able to develop a traditional pedagogical statement as originally intended. However, involving coalface teaching staff was necessary to the aims of the study and this involvement was shown to be an effective impetus for their professional development.

Values are a key component of pedagogy and play an important role in guiding teaching practices, even if they do not explicitly describe or prescribe those practices (Nind et al., 2016). Values guide decisions on what is important and how to act ethically and morally, giving a sense of direction (Apps, 1996). Awareness of and reflection upon values may, in turn, facilitate professional organisation, judgement, stability, security, and identity (Brookfield, 2006). It may also enable the development of authentic, credible, and action-oriented philosophies on teaching, pedagogical knowledge, and further development of practice (Conti, 2007; Galbraith, 2004; Galbraith & Jones, 2008). Values are intrinsically linked to student-centred theoretical frameworks, including constructivism, experiential learning, and inclusive pedagogy; specifically, these values underpin our decisions about class content and activities and evaluation of their efficacy, and can, in turn, be challenged and changed by the same processes (Antlová et al., 2015; Stentiford & Koutsouris, 2021). It should be noted that the value-based nature of the statement, along with the specific values it emphasised, may also reflect broader socialisation and contextual influences, including the performative, market-driven culture of higher education that is motivated by the goal of widening participation among of diverse students (Ball, 2015; Florian & Spratt, 2013; Tomlinson, 2018); the pressure from accrediting bodies for the incorporation of teamwork into the curriculum to help prepare graduates for work in team-based work environments; and the unique risk of our student, many of whom study online rather than on-campus, of feeling disconnected from the university (Kaufmann & Vallade, 2022). Nevertheless, we expect that our value-based statement may evolve into a pedagogical statement over time, particularly if it is used as a foundation to enhance individual growth, group professional development activities, and curriculum development on an ongoing basis (Baeten & Simons, 2014; Timperley et al., 2007; Van Driel & Berry, 2012; Watkins, 2020).

### Research question 2

Limited formal training in education may have limited participants’ awareness of and ability to express their pedagogical position, meaning that development of a collective pedagogical statement was not feasible. Staff forums were an effective means of engaging staff, particularly those not involved in previous stages. These interactions enabled us to identify and discuss any uncertainty among staff about the statement. We also encountered several barriers and enablers when applying the framework. These included the numerous competing demands placed on teaching staff, lack of awareness about the project, and ethical restrictions around the types of data that could be collected. Early and explicit communication about the benefits of developing a statement, undertaking the process as a scholarly and not a research activity, are strategies recommended to address these barriers for teaching teams looking to undertake a similar pedagogical endeavour.

### Research question 3

The collaborative approach adopted in our project was positively received by participating staff, who quantitatively and qualitatively reported valuing the opportunity to connect with colleagues and discuss teaching practices in a targeted way. Survey data suggested that participation positively and significantly impacted the confidence and competence of staff to reflect on their teaching and learning practices. These positive outcomes stand in contrast to much research that suggests that professional development activities are frequently viewed by staff as ineffective, irrelevant, and lacking both the long- and short-term impact (Opfer & Pedder, 2011). In contrast, our findings suggest that the collaborative process of developing our teaching statement was effective, relevant, and impactful for participating staff. This aligns with research that shows that activities requiring active staff participation and collaboration, and that are clearly connected to daily practice, and conducted in a safe and constructive environment are highly valued by participants (Hargreaves & O’Connor, 2017; Vreekamp et al., 2023).

### Limitations and future directions

We made a concerted effort to engage all staff in the project and give them a chance to be heard; however, despite several attempts to advertise the project through a variety of channels, response rates ranged from 29% (Stage 3) to 54% (Stage 1). This may have been due to a lack of awareness of the project or other important psychological or contextual factors. For instance, recruitment methods may have been biased against staff who have disengaged from communications about teaching and learning activities, due to workload pressures or job dissatisfaction. The timing of the project, competing tensions with busy workloads, and/or concerns about anonymity may have presented other barriers to participation. Therefore, it is possible that the themes interpreted as pedagogical values reflect participation bias of a selection of staff who are passionate about their students’ success, had the time to participate, and/or who held strong opinions (supportive or otherwise) about teaching and learning. A further limitation was that the views of teaching staff employed on casual and short-term contracts was not captured in the current study. Such staff make up a considerable proportion of the learning and teaching workforce and may offer unique insights and experiences.

Investigation into whether (and how) using this framework and the statement/s it produces influences teaching and learning professional teaching practices, conversations, departmental culture, and pedagogies of teaching teams over the long term is warranted. Longitudinal research on how having a group-level pedagogy may complement or possibly disrupt personal pedagogies, as well as the impacts on student experiences, is also warranted (Chick, 2019). Future research that reports the pedagogical stance and teaching approaches of entire teaching teams may help to further guide the development of individual teachers, student experiences, and the discipline of psychology more generally (e.g., Wagner et al., 2011).

The innovative design to capture a team’s approach to learning and teaching reported in the current study may also be applied to other disciplines within the university community. However, the statement itself is not intended to be generalisable to other teaching teams, since it reflects the unique characteristics of our university, staff and students. For example, most staff in our department are employed on permanent contracts, which likely enables a better understanding of the needs and challenges faced by our student cohort (Bell, 2021). Blended learning is the dominant teaching and learning model, given many of our students study partly or wholly online from rural, regional and remote locations. Corroborating previous research that student socialisation and participation are more difficult to achieve in online environments, and the context of our course delivery may explain the strong focus in our statement on promoting connection, accessibility, equity, and student engagement through our teaching (Ashraf et al., 2021; Kaufmann & Vallade, 2022; Shantakumari & Sajith, 2015; Sharma & Shree, 2023). We advocate for conceptualising these statements as open-ended descriptions that change in response to changes in teaching teams, and other contextual factors, over time, rather than being a summative, static document. Revision of the statement needs to occur as often as required, which may be more frequently for universities that have a more transient workforce.

## Conclusion

Our project aimed to capture the philosophies and practices of our colleagues in a collective group-level pedagogical statement. While the project did not achieve that aim, it was fruitful in that it enabled the development of an empirically-based teaching statement that captured the shared learning and teaching values of the team. We hope that the values-based statement that our project produced will encourage continued reflection, pedagogical conversations, and workplace and professional development among our colleagues, as well as provide them with a sense of place in the wider teaching community.

## Supplementary Material

Supplemental material

## Data Availability

The data that support the findings of this study are available on request from the corresponding author, LL. Student and staff data are not publicly available due to ethical restrictions.
