# Supplementary material for: Developing a shared pedagogical statement for a university psychology teaching department: is it possible?
Source: Aust J Psychol. 2026 Apr 1;78(1):2635899. doi: 10.1080/00049530.2026.2635899 (PMC13045193; doi:10.1080/00049530.2026.2635899)
Supplement: Supplemental material [file RAUP_A_2635899_SM4268.docx]

**Online Supplementary Material**

**Table 1.** Comparisons for perceptions for Stage 1 and Stage 4 online survey items.

| **Survey Item** | **D** | | ***W*** | | ***p*** | | ***d*** | |  |
| --- | --- | --- | --- | --- | --- | --- | --- | --- | --- |
| I have a clear approach/style/philosophy that guides my teaching | | +.52 | | 70.5 | | .159 | | .52 | |
| I am an effective university educator | | +.17 | | 77.0 | | .239 | | .27 | |
| I can easily describe my teaching approach/style/philosophy to others | | -.32 | | 85.0 | | .610 | | .22 | |
| I believe I can meet my learning and teaching goals | | -.17 | | 81.5 | | .687 | | .16 | |
| My current teaching practices reflects my approach/style/philosophy | | -.04 | | 88.5 | | .541 | | .04 | |

**Notes**. D = Mean difference between Stage 4 and Stage 1 responses on the respective online surveys (positive values mean greater ratings at Stage 4 compared to Stage 1); *W* = Mann Whitney-U test statistic; *p* = probability value; *d* = Cohen’s *d* effect size statistic.

**Table 2.** Associations between project participation and learning and teaching perceptions.

| **Survey Item** | ***ρ*** | ***p*** | **95% CI** | |
| --- | --- | --- | --- | --- |
|  |  |  | **LL** | **UL** |
| Your understanding of what underlies the way you teach | .52 | .043 | 0.01 | 1.00 |
| Your confidence talking about your teaching practices with your students | .54 | .035 | 0.04 | 1.00 |
| Your confidence talking about your teaching practices with the university | .54 | .035 | 0.29 | 1.00 |
| Your confidence in how you reflect on and respond to students | .49 | .052 | -0.03 | 1.00 |
| The value placed on talking about L+T practices and approaches | .30 | .174 | -0.21 | 1.00 |

**Notes**. All analyses have 11 degrees of freedom; *ρ* = Spearman’s Rank Correlation test statistic (positive values indicate direct associations); *p* = probability value; *CI =* confidence interval; *LL* = lower limit; *UL* = upper limit.
